# Editorial: Emerging talents in Frontiers in Pharmacology: Drug metabolism and transport 2022

**DOI:** 10.3389/fphar.2022.1083163

**Published:** 2022-12-07

**Authors:** Terry D. Hinds, Massimo Valoti, Yurong Lai, Thomas P. C. Dorlo, Yan Li

**Affiliations:** ^1^ Department of Pharmacology and Nutritional Sciences, Barnstable Brown Diabetes Center, Markey Cancer Center, University of Kentucky, Lexington, KY, United States; ^2^ Department of Life Sciences, University of Siena, Siena, Italy; ^3^ Department of Drug Metabolism, Gilead Sciences Inc., Foster City, CA, United States; ^4^ Department of Pharmacy, Uppsala University, Uppsala, Sweden; ^5^ School of Science, Auckland University of Technology, Auckland, New Zealand

**Keywords:** pharmacometrics, pharmacokinetics, pharmacodynamics, glucuronyl enzymes, P450 enzymes, membrane transporters, pharmacoepigenetics

Drug metabolism and transport are the key facets involving drug absorption and disposition, drug-drug interactions, and various intricate signaling pathways and protein enzyme activities. The research presented here highlights the work of emerging talented early-stage researchers across the field of drug metabolism and transport. The Research Topic covers areas such as drug metabolism and transport, endogenously formed compounds, and environmental chemicals. As part of this Research Topic, an important aspect of orally available drugs and their metabolism processes by the cytochrome P450 (CYP) isozymes were reviewed as a state-of-the-art roadmap for the metabolic fate of oral drugs (Liu et al.) The key metabolic processes for orally delivered drugs were reviewed for the assessment of current experimental systems to evaluate drug metabolism and potential interactions. A supportive manuscript on drug metabolism in this Research Topic was written by Dong et al., which investigated the effects of X-ray radiation on the pharmacokinetics of apatinib in rats and how radiation alters absorption, tissue distribution, and excretion. They found that X-rays led to significantly decreased plasma concentrations of apatinib, a tyrosine kinase inhibitor (TKI) that is used for advanced gastric cancer. The higher drug metabolic rates with X-rays are likely regulated by the expression of the cytochrome P450 or glucuronidation enzymes (Sundararaghavan et al., 2017), which can be altered by radiation therapy, indicating that drug metabolism might be affected by this treatment. In comparison, a study in rats showed that exercise induces UGT1A1 glucuronidation enzyme, which caused significantly higher plasma bilirubin levels (Hinds et al., 2020). These demonstrate that a better understanding of non-pharmacological therapies might provide unexpected outcomes through the regulation of drug metabolism pathways.

Along these lines, Bai et al. described that the regulation of CYP enzymes and drug transporters is changed under high-altitude hypoxia by the gut microbiota. Another study in the Research Topic by Li et al. characterized the therapeutic mechanism of action of *Amygdalus mongolica* oil on bleomycin-induced pulmonary fibrosis in rats. They concluded that the *Amygdalus mongolica* oil has protective effects against pulmonary fibrosis by the abundance of related intestinal flora and through the level of pulmonary fibrosis metabolites, which affected serum biomarkers of tetrahydrobiopterin, l-serine, citrulline, estradiol, glycine, serine and threonine metabolism, arginine biosynthesis and steroid hormone biosynthesis. These works suggest that the gut microbiome may also impact the pharmacokinetic profile of drugs and xenobiotics and possibly should be a consideration in the future, especially for patients administered antibiotics. Xie et al. explored the safety of colistin sulfate, an antibiotic that is used sparingly for multidrug-resistant Gram-negative infections, using population pharmacokinetics and optimized the dosing strategy for critically ill patients. They concluded that the dosage of colistin sulfate was best scaled by creatinine clearance (CrCL) and alanine aminotransferase (ALT) levels. While they did not specifically study the gut microbiome, the antibiotic might impact the liver-gut axis as indicated by ALT levels (Badmus et al., 2022). The P450 and glucuronidation enzymes regulate liver function, as shown by UGT1A1 regulation of plasma bilirubin levels (Badmus et al., 2022).

The P450 enzymes are critical mediators of drug metabolism and drug interactions. Zhao et al. showed that the cytochrome P450 enzymes CYP2J2 and CYP3A4 and ATP-binding cassette transporters BCRP/P-gp regulate the drug-drug interactions between rivaroxaban and TKI drugs. They found that the TKI drug gefitinib potently inhibited P450 isozymes, namely, CYP2J2 and CYP3A4, that metabolize rivaroxaban, a blood thinner used for the management of cardiovascular risks. They also found that the TKI drug imatinib increased systemic exposure to rivaroxaban, while sunitinib decreased its oral bioavailability in rats. While this study made progress in understanding the drug metabolism of rivaroxaban, others compared new compounds to already established drugs. Sun et al. evaluated the similarity of the monoclonal antibody QL1209 that targets HER2-positive breast cancer with Perjeta, which is an FDA-approved monoclonal antibody used in combination with trastuzumab and docetaxel for the treatment of metastatic breast cancer. They performed a randomized, double-blinded study in healthy male subjects and found that the pharmacokinetics of QL1209 were highly similar to those of Perjeta and, in addition, concluded that there was no significant difference in safety and immunogenicity between them.

The last study included in the Research Topic was by Huang et al. on lipid and polymer microbubbles (MBs) that are used as ultrasound contrast agents in clinical diagnosis. The MBs could possibly be used in ultrasound-mediated therapy due to their drug-loading capabilities. The MBs offer the advantage of tissue-specific delivery, potentially leading to increased target-site exposure to relevant drugs.

In conclusion, the research presented in this Research Topic highlights the quality and diversity of early-stage researchers across the field of drug metabolism and transport and how drug transporters and metabolizing enzymes are affected and regulate the pharmacokinetics of experimental compounds and FDA-approved drugs. The topics covered were in areas such as metabolism and transport of drugs, endogenously formed compounds, and environmental chemicals.
